# Emerging Technologies and Integrated Strategies for Microbial Detection and Control in Fresh Produce

**DOI:** 10.3390/microorganisms13071447

**Published:** 2025-06-21

**Authors:** Ayman Elbehiry, Eman Marzouk, Feras Alzaben, Abdulaziz Almuaither, Banan Abead, Mohammed Alamri, Abdulaziz M. Almuzaini, Akram Abu-Okail

**Affiliations:** 1 Department of Public Health, College of Applied Medical Sciences, Qassim University, P.O. Box 6666, Buraydah 51452, Saudi Arabia; e.marzouk@qu.edu.sa; 2 Department of Food Service, King Fahad Armed Hospital, Jeddah 23311, Saudi Arabia; 3 First Air Defense Group, Department of Nutrition, Riyadh 13624, Saudi Arabia; 4 Support Service Department, King Fahad Armed Hospital, Jeddah 23311, Saudi Arabia; 5 Family & Community Medicine Department, Prince Sultan Medical Military City, Riyadh 12233, Saudi Arabia; 6 Department of Veterinary Preventive Medicine, College of Veterinary Medicine, Qassim University, Buraydah 51452, Saudi Arabia; 7 Department of Pathology and Laboratory Diagnosis, College of Veterinary Medicine, Qassim University, Buraydah 51452, Saudi Arabia

**Keywords:** foodborne pathogens, fresh produce, microbial safety, rapid detection, public health

## Abstract

The global consumption of fresh and ready-to-eat (RTE) fruits and vegetables has surged due to increasing awareness of their nutritional benefits. However, this trend has been accompanied by a rise in foodborne illness outbreaks linked to microbial contamination. This narrative review synthesizes current knowledge on the prevalence and diversity of foodborne pathogens in fresh produce, including bacterial, viral, and fungal agents. It critically evaluates both conventional and emerging detection methods, ranging from culture-based techniques and immunoassays to advanced molecular diagnostics, biosensors, flow cytometry (FC), and hyperspectral imaging (HSI). Additionally, this review discusses cutting-edge control strategies, such as natural antifungal agents, essential oils, biocontrol methods, and non-thermal technologies like cold plasma and UV-C treatment. Emphasis is placed on sampling methodologies, sustainability, One Health perspectives, and regulatory considerations. By highlighting recent technological advances and their limitations, this review aims to support the development of integrated, effective, and safe microbial control approaches for the fresh produce supply chain.

## 1. Introduction

The global increase in consumption of fresh, raw, and RTE fruits and vegetables is largely driven by heightened public awareness of their health benefits. These foods are rich in essential vitamins, minerals, phytochemicals, and dietary fiber, and have been strongly associated with reduced risks of cardiovascular disease, type 2 diabetes, stroke, and all-cause mortality [1,2,3,4]. However, this positive dietary shift has coincided with a notable rise in foodborne illness outbreaks linked to fresh produce, primarily due to microbial contamination [5,6].

Unlike animal-based foods that typically undergo thermal processing, fruits and vegetables are often consumed raw, without any microbial inactivation steps, thereby increasing the likelihood of ingesting foodborne pathogens [7]. While bacterial pathogens such as *Escherichia coli* (*E. coli*) O157:H7, *Salmonella enterica* (*S. enterica*), and *Listeria monocytogenes* (*L. monocytogenes*) are most frequently implicated in produce-related outbreaks, non-bacterial pathogens also pose considerable risks [8,9]. Enteric viruses—particularly norovirus and hepatitis A virus—can survive on fresh produce surfaces and persist through common decontamination methods [10]. Moreover, protozoan parasites such as *Cryptosporidium* spp., *Giardia duodenalis*, and *Cyclospora cayetanensis* have been increasingly detected on fruits and vegetables, contributing to foodborne illness worldwide [11,12]. These pathogens may resist sanitizing treatments or internalize within produce tissues, complicating detection and elimination efforts. Consequently, safety strategies for fresh produce must consider a broader spectrum of pathogens—beyond bacteria—to effectively reduce foodborne disease risk.

Contamination can occur at various stages along the farm-to-fork continuum. In the preharvest phase, sources include untreated irrigation water, manure, contaminated soil, and animal intrusions [13]. During harvest and postharvest, factors such as unhygienic handling, equipment contamination, improper washing, and inadequate storage can introduce or amplify microbial risks [14,15]. Leafy greens are particularly vulnerable due to their morphology, which favors bacterial adhesion and biofilm formation [16]. The globalization of the food supply chain further complicates produce safety. Extended transport and storage durations increase the window for microbial proliferation and cross-border contamination [17]. *E. coli* and *L. monocytogenes* were responsible for additional outbreaks, including those with high morbidity and mortality [14,18].

Traditional culture-based methods, while reliable, are time-intensive and not ideal for perishable commodities. Consequently, advanced molecular and rapid detection methods have gained attention. Recent studies have increasingly evaluated techniques such as real-time PCR, multiplex PCR, and loop-mediated isothermal amplification (LAMP) for detecting foodborne pathogens in fresh produce, demonstrating high sensitivity, specificity, and potential for rapid on-site diagnostics [19,20]. Immunological assays like enzyme-linked immunosorbent assay (ELISA) and PCR–ELISA hybrids provide practical tools for routine testing [21]. Innovations in biosensor technology, including electrochemical platforms based on bacteriophage T7, offer real-time, on-site pathogen detection with high sensitivity [22]. HSI and FC provide complementary approaches for detecting viable and non-culturable pathogens on complex surfaces [23,24].

On the control side, non-thermal technologies such as cold plasma, UV-C irradiation, and antimicrobial edible coatings have shown promise for decontaminating produce without compromising sensory quality [25,26]. Plasma-activated water (PAW) has emerged as an effective and eco-friendly decontamination method [27,28]. Despite these advances, implementation barriers remain, including regulatory inconsistencies, high costs, limited infrastructure in low-income regions, and the need for specialized technical skills [29]. This review synthesizes current knowledge on microbial contamination, detection technologies, and control strategies for raw and RTE fruits and vegetables, aiming to inform future research directions and practical solutions for enhanced produce safety.

This narrative review is based on a structured search of peer-reviewed literature published between January 2000 and April 2024. Searches were conducted using scientific databases including PubMed, Web of Science, and Scopus. Keywords used in various combinations included “foodborne pathogens”, “fresh produce”, “detection methods”, “decontamination technologies”, “antimicrobial resistance”, and “microbial safety”. Articles were selected based on their relevance to microbial contamination, detection, and control strategies in raw and RTE fruits and vegetables. Additional sources were identified through manual cross-referencing of citations in key publications.

Building upon these foundations, this review offers a novel contribution by critically integrating both traditional and emerging detection and control strategies for foodborne pathogens in fresh produce with a broader and more interdisciplinary scope than previously published reviews. While Abdelshafy et al. [7] primarily focused on bacterial contamination, our work expands the discussion to include fungal spoilage, natural antifungal agents, and innovative preservation technologies. We also incorporate cutting-edge developments such as CRISPR-based diagnostics, HSI, and smart packaging solutions published between 2023 and 2025. Moreover, this review emphasizes sustainability, regulatory frameworks, and One Health perspectives, framing microbial safety not only as a technical challenge but also as an ecological and public health priority. These distinctions establish this review as a more comprehensive and forward-looking resource for addressing microbial safety in raw and RTE fruits and vegetables.

## 2. Epidemiology of Produce-Linked Foodborne Illnesses and Contamination Pathways

Unlike animal-derived foods that are typically subjected to cooking or thermal treatment, fresh produce is often consumed without any microbial inactivation step, significantly increasing the risk of exposure to pathogens. According to a review of CDC data from 2006 to 2023, *S. enterica* was responsible for 34 outbreaks linked to fresh produce in the United States, resulting in 7256 illnesses and 10 deaths. In the same period, *E. coli* O157:H7 caused 16 outbreaks with 998 illnesses and 10 deaths, while *L. monocytogenes* was implicated in 10 outbreaks that led to 303 cases and 54 deaths [7,14]. Recent studies have reported similar contamination trends in Saudi Arabia. A study by Kuddus et al. [30] analyzing RTE salads from restaurants in Hail city found significant microbial loads, including coliforms and intestinal parasites, even after multiple washings, indicating potential health risks associated with the consumption of such salads.

### 2.1. Epidemiological Trends and High-Risk Commodities

Several categories of produce are consistently linked to foodborne outbreaks. Aiyedun et al. [31] highlighted leafy greens, cucumbers, tomatoes, cantaloupes, and sprouts as the most commonly implicated commodities in both North America and Europe. Leafy greens are particularly prone to microbial contamination due to their irregular surfaces, large surface area, and ability to internalize bacteria through stomatal openings or damaged tissue [16]. Outbreak data show that produce-associated bacterial pathogens account for a significant portion of foodborne outbreaks globally. Havelaar et al. [18] estimated that 36% of all foodborne illnesses in the United States and 42% in the European Union could be attributed to contaminated fruits and vegetables. International surveillance studies further support this trend. For example, Wartha et al. [32] reported frequent detection of *L. monocytogenes*, *E. coli*, and *Staphylococcus aureus* (*S. aureus*) in RTE salads in Germany. Similarly, Bai et al. [33] found multidrug-resistant *Clostridium perfringens* in vegetable markets across China, while Oyedele et al. [34] detected toxigenic *E. coli* strains in raw produce in Nigeria. In a Canadian assessment by Zhang et al. [35], *L. monocytogenes* was found in 0.51% of fresh-cut fruits and 0.24% of vegetables, while *Salmonella* and *E. coli* O157:H7 were not detected. These results underscore the sporadic yet significant risk associated with opportunistic pathogens in produce.

Other produce types, including herbs like coriander and parsley, have also been linked to contamination. Elsafi et al. [36] identified various antibiotic-resistant bacteria, including *Enterobacter cloacae*, *Klebsiella* spp., and *Citrobacter freundii*, on leafy vegetables such as parsley and lettuce in Saudi Arabia’s eastern region. Notably, some isolates exhibited resistance to ampicillin, highlighting the potential for dissemination of resistant strains through fresh produce. In addition to bacterial contamination, the presence of fungi such as *Aspergillus* and *Penicillium* spp. on vegetables has raised concerns about mycotoxin production. Pandey et al. [37] emphasized that these fungi are common in tropical and subtropical regions, where high humidity favors their growth. Mycotoxins like ochratoxin A, produced by these fungi, pose significant health risks, including nephrotoxicity and carcinogenicity.

### 2.2. Contamination Pathways: From Preharvest to Retail

Microbial contamination of fresh produce can occur at virtually any stage along the production and distribution chain. In the preharvest environment, key contamination sources include the use of untreated or inadequately treated irrigation water, contact with contaminated soil, improperly composted manure, animal feces, and intrusion by wildlife [13,38]. A notable example is the 2006 outbreak of *E. coli* O157:H7 associated with spinach, which was traced to irrigation water contaminated by cattle feces [14]. During harvesting and handling, contamination may be introduced through the use of dirty tools, containers, gloves, or worker hands. The reuse of equipment across different fields without sufficient sanitation greatly increases the potential for cross-contamination [38,39]. Recent investigations have also highlighted the role of irrigation water quality in pathogen transfer. For example, Allende and Monaghan [40] reviewed multiple cases where *Salmonella*, *Listeria*, and pathogenic *E. coli* were introduced through water sources, particularly in regions lacking microbial water quality monitoring.

In the postharvest phase, including processing, storage, and retail display, additional microbial risks are introduced. Improper washing and cutting, unsanitary packaging conditions, and inadequate temperature control may facilitate bacterial survival and proliferation. Psychrotrophic pathogens such as *L. monocytogenes* are particularly concerning, as they are capable of growing at refrigeration temperatures [32]. Moreover, contact surfaces like conveyor belts and packaging materials can harbor bacteria and serve as long-term reservoirs if not regularly sanitized [35]. According to the findings of Olaimat and Holley [6], poorly maintained equipment and wet processing environments offer ideal conditions for biofilm formation, allowing pathogens to persist despite routine cleaning. These biofilms, especially in niches like blade assemblies and drains, can intermittently release viable cells into produce batches.

At the retail and consumer levels, produce may be stored near raw meat, subjected to improper refrigeration, or handled without adequate hygiene, further increasing the risk of contamination and cross-contact with harmful microorganisms [41,42]. Moreover, consumer behavior plays a substantial role. As shown by Redmond and Griffith [43], consumers often fail to wash produce adequately or separate it from raw animal products, creating additional routes for pathogen transfer within household kitchens.

### 2.3. Emerging Risk Factors: Climate Change, Globalization, and Antimicrobial Resistance

Emerging risk factors are reshaping the landscape of microbial safety in fresh produce. Climate change—through its effects on temperature, rainfall, and drought cycles—can influence pathogen persistence in agricultural environments [44,45]. For example, increased rainfall can result in runoff carrying fecal contaminants into irrigation water sources [17,46,47]. Simultaneously, urbanization and water scarcity have driven the reuse of untreated or poorly treated wastewater for irrigation in many developing countries, creating direct pathways for contamination [13]. A comprehensive review by Lake and Barker [48] emphasized that while precise predictions are challenging, climate-related factors such as rising temperatures and altered precipitation patterns are expected to increase the prevalence and survival of foodborne pathogens in the environment. The study called for improved modeling and surveillance systems to anticipate food safety risks under future climate conditions.

Globalization extends the distribution chain, often increasing the time between harvest and consumption. This added delay creates more opportunities for microbial proliferation and cross-border contamination. As produce travels across multiple countries and through various handlers, traceability becomes increasingly complex, and response times in outbreak scenarios are delayed [7,15,49,50]. Bintsis [51] highlighted that the expansion of global food trade has significantly elevated the risk of foodborne pathogen dissemination, including antibiotic-resistant strains, due to extended transport chains and variability in food safety standards across exporting countries.

A particularly alarming development is the growing threat of antimicrobial resistance (AMR). Multidrug-resistant *Campylobacter* spp., *L. monocytogenes*, and *S. aureus* have been isolated from vegetables in multiple countries [52,53]. These bacteria are not only more difficult to treat when infections occur, but they may also act as vectors for resistance gene transfer in the environment. Kim and Ahn [54] and Hashempour-Baltork et al. [55] both reported the presence of AMR genes in *E. coli* and *Enterococcus* strains isolated from fresh produce, highlighting the potential for produce to serve as a reservoir for resistance dissemination. Peng et al. [56] demonstrated through metagenomic analysis that mobile genetic elements carrying resistance genes—including those for beta-lactams, tetracyclines, and sulfonamides—are widespread across human, animal, and environmental sources in China. Their study revealed that fresh produce can serve as a bridge in this resistance-sharing network, underscoring the importance of a One Health approach to surveillance.

## 3. Detection Methods for Foodborne Pathogens in Fresh Produce

Ensuring the microbiological safety of fresh and RTE fruits and vegetables requires detection tools that are not only sensitive and specific but also rapid and adaptable to real-time monitoring. Effective pathogen detection also depends critically on sampling methodology, which forms the foundation for accurate analysis. Sampling of fresh produce must account for the heterogeneous distribution of pathogens across surfaces and internal tissues. For culture-based approaches, Uyttendaele et al. [57] recommend the aseptic collection of 25–50 g of produce, followed by homogenization and enrichment to optimize pathogen recovery. Swabbing and rinse techniques are commonly used for surface contamination assessment; comparative studies highlight that rinsing outperforms swabbing, especially for creviced produce [58]. In molecular methods such as qPCR and LAMP, effective removal of PCR inhibitors (e.g., polyphenols and polysaccharides) through optimized DNA extraction is essential. Miao et al. [59] demonstrated a one-step RT-qPCR capable of detecting *S. enterica* at 10 CFU/g in fresh-cut vegetables after a short enrichment, highlighting the importance of both sensitive amplification and efficient sample preparation.

For FC, Buzatu et al. [60] developed a high-sensitivity, real-time detection system and highlighted that rinsate pre-filtration and optimized staining greatly enhance accurate *L. monocytogenes* enumeration in produce matrices. HSI protocols require intact samples under controlled lighting and precise sample positioning; Lorente et al. [61] and ElMasry & Sun [62] emphasized that these parameters directly influence spoilage and microbial detection accuracy. Finally, maintaining cold-chain logistics and conducting prompt analysis (preferably within 24 h) are universally recommended to prevent microbial proliferation or die-off, as outlined by the Canadian Food Inspection Agency [63]. Appropriate sampling protocols are essential to ensure reproducibility across detection platforms and validity in assessing decontamination efficacy. Furthermore, the sensitivity and reproducibility of these methodologies vary. qPCR and RT-qPCR can detect pathogens at levels as low as 10–100 gene copies per reaction, while FC enables rapid quantification with high reproducibility in viable cell detection [60,64]. HSI shows strong spatial sensitivity but is more variable due to lighting conditions and sample geometry [61]. Clearly defined sampling protocols are therefore critical for ensuring data reliability across methods.

**Figure 1:**
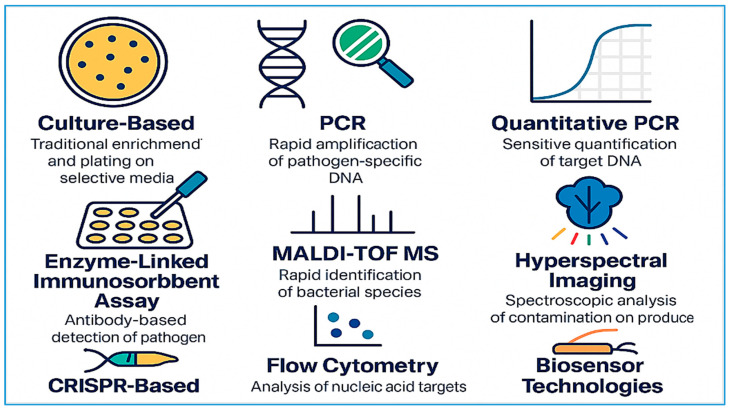
Detection methods for foodborne pathogens in fresh produce. This infographic illustrates eight major diagnostic approaches: culture-based methods, PCR, quantitative PCR (qPCR), ELISA, MALDI-TOF MS, HSI, FC, CRISPR-based detection, and biosensor technologies. Each method varies in its detection principle, sensitivity, and suitability for rapid or field-based application.

Figure 1 illustrates the diverse range of diagnostic methods currently employed to detect foodborne pathogens in fresh produce. These tools span from traditional culture-based techniques and immunological assays to advanced molecular and spectroscopic platforms. Methods such as PCR and qPCR offer rapid and specific DNA-based identification, while MALDI-TOF MS and HSI enable non-culture-based, high-resolution profiling of microbial presence. The inclusion of novel technologies like biosensors and CRISPR-based diagnostics reflects ongoing innovations aimed at enhancing detection sensitivity, speed, and field applicability. Together, these methods form a comprehensive toolkit that supports proactive microbial risk assessment and real-time safety monitoring across the fresh produce supply chain.

While traditional detection methods, such as culture-based techniques and immunological assays, have long served as the cornerstone of microbial diagnostics in fresh produce, they come with significant limitations. Culture-based methods, including selective enrichment and plating followed by biochemical or serological confirmation, remain the gold standard due to their high specificity and ability to recover live organisms for downstream analysis. They are widely accepted in regulatory frameworks and are cost-effective in terms of materials. However, these methods are labor-intensive, require skilled technicians, and are notably slow, often taking 5 to 10 days to yield actionable results. Moreover, they often fail to detect viable but non-culturable (VBNC) pathogens that may survive stress conditions such as refrigeration or sanitization [29,65].

Immunological assays, particularly enzyme-linked immunosorbent assays (ELISAs), offer quicker turnaround times and relatively simple operation. They are especially suitable for large-scale screening in routine quality control settings. Yet, their sensitivity can be compromised in complex food matrices, and they are prone to false positives or negatives due to cross-reactivity between structurally similar antigens [21]. These limitations restrict their utility when low levels of contamination must be accurately detected or when rapid results are critical.

To overcome these challenges, molecular and spectroscopic techniques have become increasingly prevalent. Quantitative PCR (qPCR) provides high sensitivity and specificity, enabling the detection of even low concentrations of pathogenic DNA in produce samples [66]. Its real-time amplification allows for quantification, which is essential for risk assessment. However, qPCR performance can be affected by inhibitors present in leafy greens or other matrix components, necessitating careful sample preparation. Droplet digital PCR (ddPCR) offers an even higher degree of accuracy and quantification by partitioning samples into thousands of micro-droplets, thus minimizing interference from inhibitors and improving reproducibility [67]. Despite their analytical advantages, both qPCR and ddPCR require high-cost instruments and reagents, as well as well-trained personnel, limiting their accessibility in low-resource settings.

MALDI-TOF MS represents a powerful proteomic tool for rapid species-level identification. It operates by generating mass spectra of bacterial proteins, which are then compared to reference libraries [68,69,70,71]. MALDI-TOF MS offers excellent throughput, low per-sample costs after setup, and minimal reagent consumption. However, its effectiveness is reduced when working directly with mixed microbial populations or environmental matrices, and spectral library limitations can impair accurate identification of novel or atypical strains.

Next-generation detection strategies include biosensor technologies, which offer portability, rapid analysis, and potential integration into automated systems. These devices employ biological recognition elements—such as antibodies, aptamers, or bacteriophages—combined with transducers that convert binding events into measurable signals. Electrochemical, magnetoelastic, and optical biosensors have shown success in detecting pathogens like *Salmonella*, *E. coli*, and *Listeria* directly from lettuce and spinach surfaces [22,72]. Still, widespread deployment remains limited by sensor robustness, matrix effects, and the need for calibration and validation.

CRISPR-based diagnostics are among the most promising innovations in molecular detection. Systems such as SHERLOCK and DETECTR leverage CRISPR-associated proteins (e.g., Cas12, Cas13) for highly specific nucleic acid detection, sometimes down to attomolar concentrations. These platforms have been adapted for food safety monitoring using lateral flow formats or fluorescence readouts [72,73]. Their advantages include programmability, speed, and compatibility with minimal infrastructure. Nonetheless, commercial adoption is still nascent due to regulatory and scalability challenges.

HSI, an advanced non-invasive technique, combines spatial and spectral data to detect microbial contamination and spoilage by analyzing unique spectral fingerprints. HSI can detect microbial growth, even in its early stages, on complex surfaces such as spinach, strawberries, and tomatoes [74,75,76,77]. However, implementation is limited by high equipment costs, the need for sophisticated data interpretation algorithms, and sensitivity to environmental lighting conditions. In parallel, the development of intelligent biosensors integrated with mobile technologies is reshaping real-time decision-making in food safety systems. Chen et al. [78] demonstrated a next-generation biosensor platform incorporating aptamer recognition, smartphone-based readout, and cloud connectivity, enabling rapid, decentralized pathogen detection. These systems align with the growing demand for on-site testing tools that are both accessible and scalable.

In summary, no single detection method is universally superior. Traditional culture-based and immunoassays remain essential for baseline and confirmatory testing, especially in regulatory and export settings. However, the increasing complexity of global food supply chains and the need for rapid, high-throughput, and field-deployable solutions underscore the importance of adopting newer molecular and spectroscopic tools. A comparative assessment of sensitivity, specificity, time-to-result, cost, technical complexity, and field applicability is crucial for selecting the appropriate platform for a given context. Blended strategies—combining traditional methods with modern technologies—may offer the most robust framework for safeguarding the microbial safety of fresh produce.

## 4. Control Strategies for Microbial Contamination in Fresh Produce

Traditional disinfection treatments such as chlorinated water washes, peracetic acid (PAA), and hydrogen peroxide rinses have historically formed the backbone of microbial control in the fresh produce industry. Chlorine-based sanitizers remain the most widely used due to their low cost, broad-spectrum efficacy, and ease of integration into large-scale washing systems [79,80]. However, they also present several critical limitations. The efficacy of chlorine is significantly reduced in the presence of organic matter, and its use can lead to the formation of potentially harmful chlorinated by-products such as trihalomethanes, raising safety and environmental concerns [81,82]. Additionally, increasing consumer and regulatory scrutiny is focused on chemical residues left on fresh produce surfaces [83].

PAA and hydrogen peroxide are viewed as residue-free alternatives with potent oxidizing capabilities. However, they may be less effective against biofilm-embedded or internalized pathogens, and improper concentrations can damage delicate fruits and vegetables, negatively impacting texture and shelf life [84,85]. From a regulatory standpoint, these sanitizers must comply with maximum residue limits and often require post-treatment rinsing, adding to operational complexity. In response to these challenges, emerging non-thermal decontamination technologies are being increasingly explored as promising alternatives that reduce or eliminate chemical use while preserving sensory and nutritional quality. These include cold plasma, UV-C, pulsed-light treatment, ozone-based sanitation, and electrolyzed water, all of which have shown substantial efficacy in inactivating a wide range of foodborne pathogens without compromising produce quality [86,87].

**Figure 2:**
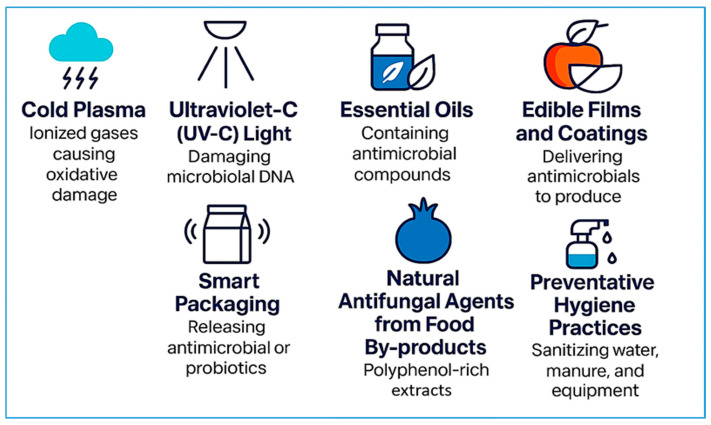
Strategies for microbial contamination control in fresh produce. This infographic highlights eight integrated approaches, including nonthermal decontamination (cold plasma and UV-C light), natural antimicrobials (essential oils and antifungal agents from pomegranate peels), advanced delivery systems (edible coatings and smart packaging), and preventative hygiene measures. These technologies work individually or synergistically to reduce microbial load while preserving produce quality.

Additionally, natural antimicrobial agents—such as essential oils, pomegranate peel extracts, and chitosan-based edible coatings—offer eco-friendly solutions with significant antimicrobial activity, although their effectiveness may vary depending on concentration, food matrix, and storage conditions [88,89]. Smart packaging systems, including oxygen scavengers and antimicrobial films, further support microbial suppression and extend product shelf life [90]. A visual overview of these principal control strategies is illustrated in Figure 2. These approaches collectively represent a multifaceted strategy to enhance microbial safety and quality in both raw and RTE fruits and vegetables, aligning with sustainability and consumer health priorities.

The following subsections provide an in-depth evaluation of these control strategies, including both emerging and established interventions. Each method is examined in terms of its mechanism of action, efficacy against foodborne pathogens, impact on produce quality, and feasibility for commercial implementation. By dissecting the strengths and limitations of individual approaches—ranging from nonthermal technologies like cold plasma and UV-C to bio-based treatments such as essential oils (EOs) and smart packaging—this review aims to offer a comprehensive, evidence-based roadmap for enhancing microbial safety in raw and RTE fruits and vegetables.

### 4.1. Cold Plasma (CP)

CP is a promising nonthermal decontamination technique utilizing ionized gases rich in reactive oxygen and nitrogen species. These species interact with bacterial cell membranes, leading to irreversible oxidative damage. The application of PAW has shown considerable efficacy in reducing microbial populations in fresh produce. For example, de Araújo Bezerra et al. [91] demonstrated that CP treatments reduced total aerobic bacteria on fresh-cut pears below the detection limit after treatment at 8 kV for 2 min. Additional studies confirmed the synergistic effects of combining CP with hydrogen peroxide, improving microbial inactivation on leafy greens and apples [25]. Mahnot et al. [92] reported that CP treatment significantly reduced microbial load on fresh-cut strawberries without compromising sensory attributes. Misra et al. [93] investigated the effects of CP generated within a sealed package on the physical quality parameters and respiration rates of strawberries. Their findings suggest that CP treatment can effectively decontaminate strawberries while retaining their quality.

### 4.2. Ultraviolet-C (UV-C) Light

UV-C light is effective against surface-bound pathogens by damaging microbial DNA. Its efficacy increases when paired with Modified Atmosphere Packaging (MAP), a technique that alters the gaseous environment inside packaging—typically by reducing oxygen and increasing carbon dioxide—to suppress microbial growth and extend shelf life. Li et al. [94] reported that UV-C + MAP reduced microbial counts in fresh-cut carrots by over 1 log CFU/g compared to control samples. In raspberries, electron beam irradiation decreased mesophilic bacterial counts by 2 log CFU/g over seven days of storage [95]. Choi et al. [96] demonstrated that combining UV-C irradiation with modified MAP significantly enhanced microbial inactivation on cherry tomatoes during cold storage. The treatment achieved a >2 log CFU/g reduction in *S. enterica* serovar Typhimurium populations and extended the product’s shelf life without compromising its sensory quality. This dual approach highlights the synergistic potential of UV-C and MAP in improving microbial safety and maintaining the freshness of fresh produce. Wang et al. [97] highlighted the potential of UV-C LED systems in reducing microbial loads on blueberries, emphasizing the importance of system design for effective surface decontamination.

### 4.3. Pulsed-Light (PL) Technology

Pulsed-light (PL) technology is a promising non-thermal decontamination technique that delivers short-duration, high-intensity pulses of broad-spectrum light (ranging from 200 to 1100 nm), including UV, visible, and infrared light. This intervention is particularly effective at inactivating surface microorganisms through mechanisms involving DNA damage, protein denaturation, and disruption of cell membranes. Its application in the fresh produce sector has gained momentum due to its minimal impact on food quality and its alignment with clean-label processing trends. Multiple studies have confirmed the efficacy of PL in reducing microbial contamination on fruits and vegetables. Bialka and Demirci [98] demonstrated that water-assisted PL treatment achieved up to 3.9 log CFU/g reductions in *E. coli* O157:H7 and *S. enterica* on raspberries without damaging the fruit. Similarly, Gómez-López et al. [99] reported a 4-log reduction of Listeria monocytogenes on packaged lettuce, while maintaining product quality.

Moreover, Ramos-Villarroel et al. [100] found that PL effectively reduced microbial counts on blueberries and strawberries when combined with water-assisted delivery, achieving significant decontamination with minimal sensory alteration. These findings are supported by Moreira et al. [101], who observed a >3 log CFU/g reduction in *Listeria innocua* on fresh-cut apples using PL at a fluence of 1.4 J/cm^2^, while maintaining color and texture stability during storage. Further reinforcing these results, Krishnamurthy et al. [102] evaluated the microbial inactivation kinetics of *L. monocytogenes* and *Salmonella* on tomatoes and mangoes and found that PL treatments achieved >4 log CFU/g reductions without any significant deterioration in visual appearance. More recently, Woldemariam et al. [103] explored PL applications in spice and dry produce safety, demonstrating its effectiveness in reducing both microbial loads and mycotoxins in red pepper powder, which underscores its broad-spectrum utility beyond conventional applications.

The effectiveness of PL treatment depends on several parameters, including fluence (total energy delivered), number of pulses, treatment distance, produce surface geometry, and light absorption characteristics. Water-assisted PL systems have proven particularly effective in mitigating surface shadowing and improving uniform exposure. The advantages of PL include minimal heat generation, rapid processing time (typically seconds), chemical-free sanitation, and compatibility with automation in postharvest processing lines. Nevertheless, challenges such as equipment cost, surface irregularities, shielding microbes, and potential minor discoloration with overexposure must be addressed to optimize commercial implementation. Despite these limitations, PL stands out as a sustainable and consumer-friendly technology for enhancing microbial safety in fresh and minimally processed produce.

### 4.4. Essential Oils (EOs)

EOs such as rosemary, eucalyptus, cinnamon, thyme, and oregano possess bioactive compounds—most notably thymol and carvacrol—that exhibit broad-spectrum antimicrobial activity against foodborne pathogens. These oils can be applied through multiple delivery methods, including direct incorporation into food matrices, coating or dipping of produce, incorporation into edible films, or use in vapor-phase treatments. For example, vapor-phase application has shown promising results in fresh produce preservation. Xylia et al. [104] reported that eucalyptus and rosemary EO vapors significantly inhibited bacterial growth and preserved cucumber quality during cold storage, suggesting this method is effective for surface decontamination without altering taste or texture [105].

In meat products, EOs are often incorporated directly at specific concentrations. Vidaković Knežević et al. [106] observed strong antilisterial effects of thyme and oregano EOs in both biofilm models and minced pork meat systems, with minimum inhibitory concentrations ranging from 0.09 to 1.78 µL/mL. Similarly, Nedić et al. [107] found that adding 0.6% oregano or thyme EO to fermented sausages effectively eliminated *L. monocytogenes* by day 14 of ripening. Notably, a lower concentration (0.3%) maintained antimicrobial action while minimizing changes to sensory quality.

To improve EO efficacy and stability, recent studies have explored synergistic applications with non-thermal technologies. When combined with ultrasound or cold plasma, the antimicrobial action of EOs is enhanced due to increased permeability of microbial membranes and better distribution within the food matrix [105]. These approaches not only reduce the required concentration of EOs but also mitigate potential impacts on flavor and aroma, making them suitable for industrial-scale use in RTE foods.

### 4.5. Edible Films and Coatings

Edible films and coatings are gaining increased attention as sustainable and efficient carriers of antimicrobial agents for fresh produce preservation. Typically derived from biopolymers such as chitosan, alginate, pectin, and starch, these biodegradable matrices are applied directly to the surface of fruits and vegetables to create semi-permeable barriers that regulate gas exchange, reduce moisture loss, and act as carriers for bioactive compounds. Kostić et al. [26] demonstrated that chitosan-based films enriched with oregano essential oil significantly inhibited *E. coli*, *Bacillus subtilis*, and *Staphylococcus epidermidis* on coated fruit surfaces, showing broad-spectrum antimicrobial efficacy. Innovations in formulation have also explored polyphenol-enhanced coatings; for instance, tannic acid/chitosan-citric acid coatings extended the shelf life of strawberries by more than 100 h and achieved over 90% bacterial growth inhibition [108]. Yang et al. [109] developed edible films composed of chitosan enriched with turmeric and green tea extracts, which were applied to strawberries. These coatings effectively suppressed the growth of *Botrytis cinerea*, delayed ripening, and preserved total phenolic content and antioxidant activity during cold storage at 4 °C, demonstrating functional and nutritional benefits.

Similarly, Rojas-Graü et al. [110] showed that alginate and gellan-based coatings supplemented with antibrowning agents maintained the visual and sensory quality of fresh-cut Fuji apples. The coatings significantly reduced enzymatic browning, preserved firmness, and slowed microbial spoilage, supporting their utility in minimally processed fruit applications. Furthermore, edible coatings are increasingly integrated with emerging preservation technologies. For example, the combination of edible coatings with pulsed-light treatment or ozonation has been reported to enhance decontamination efficiency while minimizing chemical usage and preserving organoleptic properties. These synergistic approaches are being explored to meet consumer demands for chemical-free, safe, and high-quality fresh produce. Collectively, these findings reinforce the potential of edible films and coatings as environmentally friendly, multifunctional strategies to enhance the microbial safety, quality, and shelf life of raw and RTE fruits and vegetables.

### 4.6. Electrolyzed Water

Electrolyzed water (EW), especially slightly acidic electrolyzed water (SAEW) and neutral electrolyzed oxidizing water, has emerged as an environmentally friendly and highly effective method for sanitizing fresh produce. These fluids exhibit strong antimicrobial action due to hypochlorous acid and other active chlorine species, which damage microbial membranes, proteins, and DNA without leaving harmful residues or byproducts. A proteomic study by Chang et al. [111] (equivalent here to a recently conducted SAEW proteomic analysis) revealed that SAEW treatment induced oxidative stress and denatured ribosomal proteins in *L. monocytogenes*, subsequently leading to VBNC states—an important consideration for food safety monitoring.

In terms of practical decontamination, Guentzel et al. [112] (similar to early EW-spinach washing trials) found that using neutral EW (approx. 100 ppm free chlorine) with mild agitation reduced *E. coli*, *Salmonella enterica*, and *L. monocytogenes* by 4–5 log CFU/g on spinach and lettuce in a water bath. Another impactful study by Afari et al. [113] reported that automated produce washing using neutral-pH EW (155 mg/L free chlorine, pH 7.5) achieved reductions of 4.2–5.9 log CFU/g for *E. coli* O157:H7 and 4.6–6.0 log CFU/g for *Salmonella typhimurium* across romaine lettuce, iceberg lettuce, and tomatoes.

Moreover, Hamidi et al. [114] demonstrated that combining neutral EW with PAA produced synergistic effects, achieving >2.5 log reductions in *E. coli*, *Salmonella*, and *L. monocytogenes* on mixed produce and meat samples. Beyond planktonic cells, SAEW is also effective against biofilms: Chang et al. [111], in a recent ultrasonication-assisted SAEW study, showed >1.5 log CFU/mL reduction of Listeria and *E. coli* biofilms on stainless steel surfaces, illustrating the benefit of combining EW with physical agitation.

Despite its advantages, EW’s effectiveness can be influenced by factors such as organic matter load, free chlorine concentration, and pH. Over-dilution or inappropriate application may lead to insufficient microbial inactivation or induction of viable-but-nonculturable states. Therefore, optimizing operational parameters and integrating EW with other physical or chemical treatments (e.g., ultrasonication or agitation) can improve its efficacy. In conclusion, EW represents a safe, effective, and sustainable alternative to traditional sanitizers for fresh produce, with broad applicability in both small- and large-scale operations.

### 4.7. Ozone Treatment

Ozone treatment has become an effective non-thermal method for microbial decontamination of fresh produce, applied in either gaseous or aqueous forms. It operates through the strong oxidative capacity of ozone, which damages microbial cell membranes, proteins, and nucleic acids, ultimately leading to cell death. One of the advantages of ozone is that it rapidly decomposes into oxygen, leaving no harmful residues, making it particularly suitable for organic and sustainable food processing systems. Aqueous ozone (ozonated water) has been widely studied for its use in postharvest washing of fruits and vegetables. A comprehensive meta-analysis by Hong et al. [115] evaluated multiple ozone application methods and reported that sparging or bubbling ozone through water could achieve 2–4 log CFU/g reductions in *Salmonella*, *L. monocytogenes*, and *E. coli* on produce like spinach, lettuce, and berries. Their review emphasized that treatment efficiency depends on factors such as water temperature, pH, ozone concentration, and contact time.

Gibson et al. [116] demonstrated that ozonated water at 13 mg/L significantly reduced *Salmonella enterica*, *Listeria innocua*, and *E. coli* on tomatoes and leafy greens by more than 4 log CFU/g, without altering texture or flavor. Their study also highlighted that ozone exposure times between 5 and 10 min are generally sufficient for effective microbial reduction. Gaseous ozone is also utilized, particularly in packaging and storage settings. Macías-Gallardo et al. [117] investigated the use of continuous low-dose gaseous ozone (0.3–1.0 ppm) during cold storage of strawberries. They found a significant reduction in fungal growth and maintenance of fruit firmness and color at 0.3 ppm, although higher concentrations led to mild oxidative changes. This illustrates the need for precise control to balance microbial safety and product quality.

Ozone treatment has demonstrated antifungal efficacy against key spoilage organisms such as *Botrytis cinerea* and *Penicillium expansum*. Luo et al. [118] showed that daily exposure to 79 ppm gaseous ozone significantly reduced fungal growth on kiwifruit while preserving quality. Similarly, Ozkan et al. [119] reported effective suppression of fungal decay on table grapes using 1.5 ppm ozone gas during storage. When combined with interventions like MAP or ultrasonication, ozone further enhances microbial inactivation. Its residue-free nature and broad-spectrum activity make ozone an eco-friendly option for decontaminating minimally processed and organic produce, provided application parameters are carefully optimized.

### 4.8. Hurdle Technology

Combining multiple treatments such as UV-C light, cold plasma, essential oils, and antimicrobial coatings can offer synergistic benefits. Abdelshafy et al. [120] reported that hurdle technologies significantly enhanced disinfection performance while maintaining sensory quality, especially for pathogens like *Salmonella typhimurium* and *L. monocytogenes* on leafy greens. Zhou et al. [121] demonstrated the application of power ultrasound combined with chemical sanitizers (chlorine and peroxyacetic acid) on grape tomatoes, romaine lettuce, and spinach. This combination achieved a microbial reduction of up to 3.99 log CFU/g for *L. monocytogenes* and 3.62 log CFU/g for *Salmonella* Newport, significantly outperforming the use of sanitizers or ultrasound alone.

Liu et al. [122] applied a novel physical hurdle approach by combining a low-voltage electrostatic field with MAP to extend the shelf life of button mushrooms. This combination reduced microbial spoilage while preserving sensory and nutritional quality throughout cold storage. In another study, Melhem et al. [123] conducted a meta-analysis assessing the synergy of high-pressure processing and natural antimicrobials, such as bacteriocins. While primarily applied to cured meats, the findings provide strong evidence for the cross-applicability of this hurdle strategy to fresh produce, especially for the control of *L. monocytogenes* and other resilient pathogens. Together, these studies underscore the value of combining complementary interventions to overcome microbial resistance mechanisms and preserve product quality, aligning with consumer demand for safe, minimally processed produce.

### 4.9. Smart Packaging and Responsive Films

Smart packaging technologies represent an innovative approach to improving the microbial safety, shelf life, and consumer confidence in fresh and minimally processed produce. These systems utilize responsive materials, biosensors, or visual indicators to monitor spoilage-related changes such as gas emission, pH fluctuation, or microbial growth. Mahović Poljaček et al. [124] demonstrated the development of starch-based films enhanced with bacterial nanocellulose and natural dyes that change color with pH shifts, offering both antimicrobial activity and freshness indicators. Such smart films are promising for future applications in produce monitoring and consumer safety.

Wu et al. [125] developed high-sensitivity intelligent packaging films incorporating rose anthocyanins that provide clear, colorimetric changes in response to pH variation. These films, tested on shrimp, effectively indicated spoilage progression and demonstrated potential adaptability to monitor the freshness of produce through pH-sensitive reactions. Zhang et al. [126] introduced biopolymer-based intelligent packaging integrated with natural colorimetric sensors for real-time food quality assessment. Their packaging system, based on biodegradable matrices, successfully detected spoilage-related VOCs and pH changes in perishable foods, aligning with sustainability goals in food safety monitoring. Additionally, Palanisamy et al. [127] reviewed recent advances in intelligent food packaging systems that combine biodegradable polymer matrices with natural sensors for detecting spoilage-related changes such as gas emission or temperature shifts. Their study emphasized how these smart films reduce food waste and improve food safety across the produce supply chain.

In a related innovation, Douaki et al. [128] developed battery-free, stretchable, and autonomous smart packaging that enables real-time monitoring of freshness through closed-loop sensing and release mechanisms. These systems operate without external power sources, making them suitable for wireless freshness tracking of high-value produce in cold chain logistics. Together, these findings highlight the growing utility of smart packaging as an effective strategy to reduce spoilage, monitor quality, and deliver intelligent, eco-friendly solutions tailored to fresh produce safety.

### 4.10. Application of Natural Antifungal Agents

Fungal spoilage is a major concern in the preservation of fresh produce, contributing to significant postharvest losses and reduced consumer safety. The application of natural antifungal agents, particularly those derived from plant extracts, EOs, and probiotic metabolites, has gained momentum as a safer alternative to synthetic fungicides. Nawaz et al. [129] demonstrated that polyphenol-rich extracts from pomegranate peels exhibited potent antifungal activity against a broad spectrum of postharvest pathogens, including *Aspergillus niger*, *Penicillium expansum*, and *Colletotrichum gloeosporioides*. The n-hexane fraction, containing bioactive compounds like nobiletin and salidroside, was found to be the most effective. Boubrik et al. [130] further supported the antifungal efficacy of EOs by demonstrating that Cinnamomum cassia essential oil inhibited the growth of spoilage yeasts and molds such as *Saccharomyces cerevisiae* and *Acremonium* spp. Their study revealed minimum inhibitory concentrations of 6.25% and 3.125%, respectively, underscoring the oil’s potential as a natural preservative in fruit-based applications.

Similarly, Aljuhani et al. [131] assessed the antifungal activity of methanolic extracts of *Carica papaya* fruits against *Microsporum canis*. The extract produced a 37 mm zone of inhibition and an MIC of 1000 µg/mL. Phytochemical analysis identified compounds like xanthosine and decanoic acid as contributors to its antifungal action, highlighting the potential use of papaya extracts in antifungal formulations for produce protection. Additionally, Vasundaradevi et al. [132] isolated a strain of *Lactiplantibacillus argentoratensis* from tropical fruits that exhibited potent antifungal activity against *Fusarium oxysporum*. The cell-free supernatant from this probiotic strain inhibited spore germination and reduced fungal biomass by 94%, with citric, lactic, and malic acids identified as key antifungal metabolites. These findings support the development of bio-based fungistatic treatments for postharvest disease management.

Despite their natural origin, some plant-derived compounds and EOs may pose risks of cytotoxicity, allergenicity, or organoleptic alterations at higher concentrations. For example, certain phenolic constituents—such as cinnamaldehyde and eugenol—have been associated with skin sensitization or irritation in sensitive individuals [133]. Moreover, the Generally Recognized As Safe status does not preclude the need for dose optimization and regulatory evaluation in food applications [134]. Therefore, while these natural agents offer promising residue-free antifungal solutions, safety profiling and controlled application are essential for consumer protection. Collectively, these studies demonstrate that natural antifungal agents—ranging from botanical extracts to microbial metabolites—offer sustainable, residue-free alternatives for controlling fungal contamination in fresh and minimally processed produce.

### 4.11. Preventative Measures

Preventing bacterial and fungal contamination in fresh produce requires an integrated approach that begins at the farm level and continues throughout postharvest handling, packaging, and distribution. This “farm-to-fork” strategy is widely recognized as essential for reducing microbial risks across the fresh produce supply chain [135]. At the preharvest stage, adherence to Good Agricultural Practices (GAPs)—such as using clean irrigation water, proper composting of manure, crop rotation, and selecting disease-resistant cultivars—is critical for minimizing the introduction of pathogens and fungal colonization [136]. For instance, irrigation timing and water quality have been shown to significantly influence contamination levels, with studies reporting higher prevalence of *L. monocytogenes* in fields irrigated shortly before harvest [137]. Public outreach resources, such as those provided by the University of Nevada, Reno Extension, emphasize that GAPs tailored to local environmental conditions and farming practices are essential to prevent contamination at the source [138].

In postharvest handling, microbial contamination may arise from unsanitary equipment, inadequate worker hygiene, or improper washing and cooling procedures. While chlorine-based sanitizers are widely used, concerns over chemical residues and by-product formation have led to the adoption of alternatives such as EW and ozone treatments. These eco-friendly methods reduce microbial load without compromising the safety or sensory quality of fresh produce. Biopreservation methods are gaining prominence, particularly the use of lactic acid bacteria and their metabolic by-products, such as organic acids, hydrogen peroxide, and bacteriocins. These compounds exert antifungal effects by lowering pH, damaging fungal membranes, and inhibiting spore germination. Recent studies have demonstrated that lactic acid bacteria exhibit strong antifungal activity, effectively extending the shelf life of fresh fruits and vegetables while preserving sensory quality [139,140].

In addition, biological control agents (BCAs) such as antagonistic yeasts and beneficial bacteria offer a natural alternative to chemical fungicides. These BCAs function through mechanisms like competition for nutrients and space, production of antifungal enzymes, and interference with fungal cell signaling. As reviewed by Nunes [141], the application of BCAs has shown considerable efficacy in controlling postharvest diseases in citrus, grapes, apples, and other high-value produce. Finally, innovative packaging solutions such as MAP and active packaging can create hostile environments for fungal proliferation. These systems regulate internal oxygen and moisture levels, often incorporating antimicrobial films or coatings that further suppress microbial activity. Together, these preventative measures form a robust and sustainable framework for microbial risk reduction in fresh produce supply chains, contributing to safer, longer-lasting products from farm to consumer.

## 5. Implementation Challenges and Future Directions for Pathogen Control in Fresh Produce

Despite significant advances in pathogen detection and control technologies for fresh and RTE produce, practical implementation remains limited by multifaceted challenges (Table 1). Barriers to adoption include economic constraints, regulatory inconsistencies, technical complexity, and infrastructural deficiencies, particularly in low- and middle-income countries (LMICs). High equipment and maintenance costs, along with limited scalability, hinder the widespread use of advanced diagnostic platforms (Table 1). Technologies such as real-time PCR, ddPCR, MALDI-TOF MS, and HSI offer superior sensitivity and specificity; however, they require expensive instrumentation, specialized consumables, and technically skilled personnel. Ferone et al. [29] emphasized that these tools are largely confined to high-resource laboratory environments due to their operational demands. Abdelshafy et al. [7] similarly observed that implementation challenges are especially acute in LMICs, where laboratory infrastructure is insufficient despite frequent foodborne disease outbreaks.

A further obstacle is the lack of harmonized and enforceable regulatory frameworks for produce safety. In the United States, for instance, the FDA encourages voluntary adherence to GAPs and Good Handling Practices (GHPs), but enforcement remains inconsistent and often decentralized [142]. In many developing countries, food safety regulations are outdated, poorly enforced, or entirely absent, while food surveillance programs suffer from chronic underfunding. In response, the WHO [143] has advocated for global policy coordination, emphasizing the strengthening of surveillance systems and AMR monitoring in foodborne pathogens.

Infrastructure limitations in rural and peri-urban regions—such as lack of access to clean water, refrigerated transportation, sanitary packaging environments, and reliable cold storage—further compound microbial risks. These conditions are particularly problematic for high-risk commodities like leafy greens and soft-skinned fruits, which possess intricate surface morphologies that favor microbial adhesion and biofilm formation [16]. De Araújo Bezerra et al. [91] also highlighted that the absence of continuous electricity and cold chain systems in tropical climates increases postharvest vulnerability to contamination.

Another key challenge is the workforce skill gap. The effective use of advanced technologies such as MALDI-TOF MS, next-generation sequencing, and biosensor systems requires trained microbiologists, molecular biologists, and bioinformaticians. However, many laboratories—especially in resource-limited settings—lack personnel with the expertise to process and interpret complex datasets. Ferone et al. [29] noted that this deficit significantly impedes the deployment of rapid diagnostic tools at the point of need. Moreover, there remains a substantial disconnect between laboratory innovation and commercial application. Promising technologies, such as CRISPR-based diagnostics and magnetoelastic biosensors, have shown strong performance in controlled trials but face hurdles in cost-effective validation, regulatory approval, and integration into existing food safety frameworks [144]. As Abdelshafy et al. [7] stressed, bridging this translational gap will require robust public–private partnerships and sustained investment.

To overcome these systemic challenges, a shift toward integrated, science-driven strategies is essential. Investment in mobile, low-cost diagnostic tools—such as portable PCR units and lateral flow assays—should be paired with infrastructural development, particularly in rural agricultural hubs. Concurrently, national and regional capacity-building initiatives, including workforce training programs and food safety certification schemes, can address skill shortages and promote regulatory compliance. Furthermore, policy harmonization guided by the One Health approach will be pivotal in addressing interconnected risks across human, animal, and environmental health domains.

A paradigm shift is urgently needed—one that prioritizes proactive, systems-based prevention over reactive responses. This transition should be grounded in scientific evidence, sustainability principles, and cross-sectoral collaboration. Ultimately, global cooperation, inclusive policies, and strategic investment will be indispensable to ensure the microbiological safety of fresh produce across all socioeconomic contexts.

## 6. Recommendations and Future Directions for Enhancing Microbial Safety of Fresh Produce

Enhancing the microbial safety of fresh and RTE fruits and vegetables requires a coordinated, systems-based approach that integrates regulatory, technological, and educational interventions (Table 2). Key priorities include the standardization of preventive practices, the advancement of accessible technologies, the harmonization of regulatory oversight, and capacity-building across the supply chain.

A critical first step is the widespread enforcement and harmonization of GAPs, GHPs, and hygiene protocols. While agencies like the U.S. FDA and USDA provide guidance for voluntary adoption, uptake remains uneven, especially in low-resource contexts [145,146,147]. Integrating third-party audits and routine monitoring—focused on irrigation water quality, manure management, field sanitation, and postharvest infrastructure—is essential for ensuring compliance and reducing pathogen risk [57].

Targeted investment in cost-effective, scalable technologies is vital for low- and middle-income countries. Non-thermal decontamination methods such as PAW, UV-C light, and essential oil-based nanoemulsions offer effective microbial control while preserving produce quality [91,105]. Meanwhile, field-deployable diagnostic tools—such as multiplex qPCR and biosensors—should be prioritized for rapid, on-site detection of foodborne pathogens [148,149]. To support global coordination, a unified regulatory framework aligned with Codex Alimentarius and backed by the WHO, FAO, and OIE is needed. Integrating whole-genome sequencing and resistome surveillance into national monitoring programs will strengthen AMR detection and response as part of a One Health strategy [143].

Human capital development is equally critical. Training programs for food handlers, laboratory technicians, and regulatory staff should focus on sanitation, diagnostics, and AMR mitigation. Competency-based curricula and applied technical workshops—delivered through partnerships with academic institutions and industry—can enhance readiness to deploy advanced tools such as MALDI-TOF MS and CRISPR-based diagnostics [55]. To accelerate translation of research into practice, funding should support prototype development, field validation, and regulatory approval of emerging technologies. Public–private partnerships can scale promising solutions, such as antimicrobial edible coatings and HSI-integrated screening systems [7].

Looking ahead, the future of produce safety will be shaped by smart surveillance networks. Artificial intelligence, IoT-enabled sensors, and blockchain platforms will enhance traceability and enable real-time risk assessments [150,151]. Emerging efforts are already demonstrating the value of integrating CRISPR diagnostics with portable sequencing platforms for strain-level detection in field settings [152]. These technologies—combined with mobile tracking apps and blockchain-verified distribution chains—are being piloted in agricultural hubs for real-time monitoring of leafy greens and other high-risk produce. In sum, ensuring the microbial safety of fresh produce demands cross-sectoral collaboration, innovation, and sustained investment. Only through integrated strategies that unite science, technology, policy, and education can we build resilient, safe food systems for the future.

## 7. Conclusions

The microbial safety of fresh and RTE fruits and vegetables is a complex challenge that spans preharvest practices, postharvest handling, detection technologies, and intervention strategies. While traditional microbiological methods remain important, the integration of rapid, sensitive, and field-deployable tools—such as CRISPR-based diagnostics, biosensors, and MALDI-TOF MS—is critical to modern surveillance. Natural antifungal agents and non-thermal treatments show great promise as eco-friendly control strategies but require further standardization and safety assessments, particularly regarding toxicity and allergenicity. A major advancement presented in this review is the inclusion of comprehensive sampling methodologies and the critical evaluation of method sensitivity, specificity, and reproducibility. Future research must address scalability, cost-effectiveness, and policy harmonization under the One Health framework. A multifaceted and interdisciplinary approach is essential to safeguard public health and meet the growing demand for safe, minimally processed produce.

## Figures and Tables

**Table 1:** Key implementation challenges and strategic solutions for pathogen control in fresh produce.

Challenge Category	Specific Barriers	Strategic Solutions/Future Directions
Economic Constraints	High cost of advanced detection technologies (e.g., PCR, MALDI-TOF MS)	Investment in low-cost portable diagnostics (e.g., lateral flow assays, portable PCR)
Technical Limitations	Need for skilled personnel; complex data interpretation	Capacity-building through training programs; simplified, user-friendly diagnostic platforms
Regulatory Gaps	Fragmented or outdated food safety regulations, especially in LMICs	Policy harmonization guided by One Health approach; enhanced enforcement and international cooperation
Infrastructure Deficiencies	Lack of clean water, refrigeration, hygienic packaging, and cold chains	Development of basic infrastructure in rural/agricultural zones; support for smallholder supply chains
Laboratory–Field Disconnect	Technologies remain confined to labs; limited real-world application	Promote public–private partnerships; validation of tools in field settings; scale-up of innovations
Environmental Conditions	Climate variability, poor electricity access, and tropical humidity exacerbate risks	Deploy solar-powered cooling, weather-resilient logistics, and robust packaging systems
Workforce Shortage	Limited availability of microbiologists, bioinformaticians, and food safety experts	Establish food safety certification programs; invest in STEM education and international knowledge transfer

**Table 2:** Strategic actions to enhance microbial safety of fresh produce.

Strategic Focus Area	Action Item	Expected Outcome
Preventive Frameworks	Enforce GAPs, GHPs, and sanitation standards with third-party audits	Improved baseline hygiene and reduced contamination risk
Technology Innovation	Develop and deploy cost-effective tools (e.g., PAW, UV-C, qPCR, biosensors)	Real-time pathogen detection and non-thermal decontamination
Regulatory Harmonization	Adopt Codex-based global standards supported by WHO/FAO/OIE	Consistent safety benchmarks and enhanced AMR surveillance
Capacity Building	Train personnel in sanitation, diagnostics, and AMR control	Workforce readiness and effective tool deployment
Translational Research	Fund prototype development and field validation	Accelerated adoption of innovations
Public-Private Partnerships	Support scale-up of coatings, HSI, and smart packaging	Commercialization of pilot technologies
Smart Surveillance	Integrate AI, IoT, blockchain, and CRISPR-based tools	Real-time traceability and rapid outbreak response

## Data Availability

No new data were created or analyzed in this study. Data sharing is not applicable to this article.
